# Uniform convergence guarantees for the deep Ritz method for nonlinear problems

**DOI:** 10.1186/s13662-022-03722-8

**Published:** 2022-07-15

**Authors:** Patrick Dondl, Johannes Müller, Marius Zeinhofer

**Affiliations:** 1 Department of Applied Mathematics, University of Freiburg, Hermann-Herder-Straße 10, 79104 Freiburg i. Br., Germany; 2 Max Planck Institute for Mathematics in the Sciences, Inselstraße 22, 04103 Leipzig, Germany; 3 Simula Research Laboratory, Department of Numerical Analysis and Scientific Computing, Kristian Augusts Gate 23, 0164 Oslo, Norway

**Keywords:** Calculus of variations, Nonlinear problems, Ritz method, Boundary penalty method, Neural networks

## Abstract

We provide convergence guarantees for the Deep Ritz Method for abstract variational energies. Our results cover nonlinear variational problems such as the *p*-Laplace equation or the Modica–Mortola energy with essential or natural boundary conditions. Under additional assumptions, we show that the convergence is uniform across bounded families of right-hand sides.

## Introduction

The idea of the Deep Ritz Method is to use variational energies as an objective function for neural network training to obtain a finite-dimensional optimization problem that allows solving the underlying partial differential equation approximately. The idea of deriving a finite-dimensional optimization problem from variational energies dates back to Ritz [28], was widely popularized in the context of finite element methods (see, e.g., Braess [4]), and was recently revived by E and Yu [13] using deep neural networks. In the following, we give a more thorough introduction to the Deep Ritz Method. Let $\Omega \subseteq \mathbb{R}^{d}$ be a bounded domain and consider the variational energy corresponding to the Lagrangian *L* and a force *f*, namely

1 $$ E\colon X\to \mathbb{R}, \qquad E(u) = \int _{\Omega }L\bigl(\nabla u(x), u(x), x\bigr) - f(x)u(x) \,\mathrm{d}x, $$

defined on a suitable function space *X*, usually a Sobolev space $W^{1,p}(\Omega )$. One is typically interested in minimizers of *E* on subsets $U\subseteq X$ where *U* encodes further physical constraints, such as boundary conditions. Here, we consider either unconstrained problems or zero Dirichlet boundary conditions and use the notation $U=X_{0}$ for the latter case. In other words, for zero boundary conditions, one aims to find

2 $$ u \in \underset{v\in X_{0}}{\operatorname{argmin}} \int _{\Omega }L\bigl( \nabla v(x),v(x),x\bigr) - f(x)v(x) \,\mathrm{d}x. $$

To solve such a minimization problem numerically, the idea dating back to Ritz [28] is to use a parametric ansatz class

3 $$ A := \bigl\{ u_{\theta }\in X \mid \theta \in \Theta \subseteq \mathbb{R}^{P} \bigr\} \subseteq U $$

and to consider the finite-dimensional minimization problem of finding

$$ \theta ^{*} \in \underset{\theta \in \Theta}{\operatorname{argmin}} \int _{\Omega }L\bigl(\nabla v_{\theta}(x),v_{\theta}(x),x \bigr) - f(x)v_{ \theta}(x)\,\mathrm{d}x $$

which can be approached by different strategies, depending on the class *A*. For instance, if *A* is chosen to be a finite element ansatz space or polynomials and the structure of *E* is simple enough, one uses optimality conditions to solve this problem.

In this manuscript, we focus on ansatz classes that are given through (deep) neural networks. When choosing such ansatz functions, the method is known as the Deep Ritz Method and was recently proposed by E and Yu [13]. Neural network type ansatz functions possess a parametric form as in (3), however, it is difficult to impose zero boundary conditions on the ansatz class *A*. To circumvent this problem, one can use a penalty approach, relaxing the energy to the full space, but penalizing the violation of zero boundary conditions, to include these. This means that for a penalization parameter $\lambda > 0$ one aims to find

4 $$ \theta _{\lambda}^{*} \in \underset{\theta \in \Theta}{\operatorname{argmin}} \int _{\Omega }L\bigl( \nabla v_{\theta}(x),v_{\theta}(x),x \bigr) - f(x)v_{\theta}(x)\,\mathrm{d}x + \lambda \int _{\partial \Omega}v^{2}_{\theta}\,\mathrm{d}s. $$

The idea of using neural networks for the approximate solution of PDEs can be traced back at least to the works of Lee and Kang [21], Dissanayake and Phan-Thien [10], Takeuchi and Kosugi [32], Lagaris et al. [20]. Since the recent successful application of neural network based methods to stationary and instationary PDEs by E et al. [12], E and Yu [13], Sirignano and Spiliopoulos [30], there is an ever growing body of theoretical works contributing to the understanding of these approaches. For a collection of the different methods, we refer to the overview articles by Beck et al. [3], Han et al. [15].

The error in the Deep Ritz Method, which decomposes into an approximation, optimization, and generalization terms, has been studied by Luo and Yang [25], Xu [34], Duan et al. [11], Hong et al. [17], Jiao et al. [18], Lu et al. [23], Lu et al. [24], Müller and Zeinhofer [26]. However, those works either consider non-essential boundary conditions or they require a term with a positive potential, apart from Müller and Zeinhofer [26]. This excludes the prototypical Poisson equation, which was originally treated by the Deep Ritz Method by E and Yu [13]. More importantly, those works only study linear problems, which excludes many important applications.

In this work, we thus study the convergence of the Deep Ritz Method when a sequence of growing ansatz classes $(A_{n})_{n\in \mathbb{N}}$, given through parameter sets $\Theta _{n}$ and a penalization of growing strength $(\lambda _{n})_{n\in \mathbb{N}}$ with $\lambda _{n} \nearrow \infty $, is used in the optimization problem (4) with more modest assumptions on *L*, *f*, and Ω.

Denote a sequence of (almost) minimizing parameters of problem (4) with parameter set $\Theta _{n}$ and penalization $\lambda _{n}$ by $\theta _{n}$. We then see that under mild assumptions on $(A_{n})_{n\in \mathbb{N}}$ and *E*, the sequence $(u_{\theta _{n}})_{n\in \mathbb{N}}$ of (almost) minimizers converges weakly in *X* to the solution of the continuous problem, see Theorem 7 in Sect. 3. We then strengthen this result in Sect. 4 where we show that the aforementioned convergence is uniform across certain bounded families of right-hand sides *f*, see Theorem 12. This means that a fixed number of degrees of freedom in the ansatz class can be used independently of the right-hand side to achieve a given accuracy. Alternatively, given a discretization of the space of right-hand sides, one may discretize the solution operator that maps *f* to the minimizer *u* of (2) and still obtain a convergence guarantee (although this is not necessarily a viable numerical approach).

To the best of our knowledge, our results currently comprise the only convergence guarantees for the Deep Ritz Method for nonlinear problems. However, since we prove these results using Γ-convergence methods, no rates of convergence are obtained – as mentioned above, for linear elliptic equations some error decay estimates are known. Our results also do not provide insight into the finite-dimensional optimization problem (4) which is a challenging problem in its own right, see, for instance, Wang et al. [33], Courte and Zeinhofer [8]. However, they guarantee that given one is able to solve (4) to a reasonable accuracy, one is approaching the solution of the continuous problem (2).

Our results are formulated for neural network type ansatz functions due to the current interest in using these in numerical simulations, yet other choices are possible. For instance, our results do apply directly to finite element functions.

The remainder of this work is organized as follows. Section 2 discusses some preliminaries and the used notation. The main results, namely Γ-convergence and uniformity of convergence are provided in Sects. 3 and 4, respectively. Finally, in Sect. 5 we discuss how the *p*-Laplace and a phase field model fit into our general framework.

## Notation and preliminaries

We fix our notation and present the tools that our analysis relies on.

### Notation of Sobolev spaces and Friedrich’s inequality

We denote the space of functions on $\Omega \subseteq \mathbb{R}^{d}$ that are integrable in the *p*th power by $L^{p}(\Omega )$, where we assume that $p\in [1,\infty )$. Endowed with

$$ \lVert u\rVert _{L^{p}(\Omega )}^{p} := \int _{\Omega }\lvert u \rvert ^{p}\,\mathrm{d}x, $$

this is a Banach space, i.e., a complete normed space. If *u* is a multivariate function with values in $\mathbb{R}^{m}$, we interpret $\lvert \cdot \rvert $ as the Euclidean norm. We denote the subspace of $L^{p}(\Omega )$ of functions with weak derivatives up to order *k* in $L^{p}(\Omega )$ by $W^{k,p}(\Omega )$, which is a Banach space with the norm

$$ \lVert u\rVert _{W^{k,p}(\Omega )}^{p} := \sum _{l=0}^{k} \bigl\lVert D^{l} u \bigr\rVert _{L^{p}(\Omega )}^{p}. $$

This space is called a *Sobolev space* and we denote its dual space, i.e., the space consisting of all bounded and linear functionals on $W^{k,p}(\Omega )$ by $W^{k,p}(\Omega )^{\ast}$. The closure of all compactly supported smooth functions $\mathcal {C}_{c}^{\infty}(\Omega )$ in $W^{k,p}(\Omega )$ is denoted by $W^{k,p}_{0}(\Omega )$. It is well known that if Ω has a Lipschitz continuous boundary the operator that restricts a Lipschitz continuous function on Ω̅ to the boundary admits a linear and bounded extension $\operatorname{tr}\colon W^{1,p}(\Omega )\to L^{p}(\partial \Omega )$. This operator is called the *trace operator* and its kernel is precisely $W^{1,p}_{0}(\Omega )$. Further, we write $\lVert u\rVert _{L^{p}(\partial \Omega )}$ whenever we mean $\lVert \operatorname{tr}(u)\rVert _{L^{p}(\partial \Omega )}$. In the following we mostly work with the case $p=2$ and write $H^{k}_{(0)}(\Omega )$ instead of $W^{k,2}_{(0)}(\Omega )$.

In order to study the boundary penalty method, we use the Friedrich inequality which states that the $L^{p}(\Omega )$ norm of a function can be estimated by the norm of its gradient and boundary values. We refer to Gräser [14] for a proof.

#### Proposition 1

(Friedrich’s inequality)

*Let* $\Omega \subseteq \mathbb{R}^{d}$ *be a bounded and open set with Lipschitz boundary* *∂*Ω *and* $p\in (1,\infty )$. *Then there exists a constant* $c>0$ *such that*

5 $$ \lVert u\rVert _{W^{1,p}(\Omega )}^{p}\le c^{p} \cdot \bigl(\lVert \nabla u\rVert _{L^{p}(\Omega )}^{p} + \lVert u\rVert _{L^{p}( \partial \Omega )}^{p} \bigr) \quad \textit{for all } u\in W^{1,p}( \Omega ). $$

### Neural networks

Here we introduce our notation for the functions represented by a feedforward neural network. Consider natural numbers $d, m, L, N_{0}, \dots , N_{L}\in \mathbb{N}$ and let

$$ \theta = \bigl((A_{1}, b_{1}), \dots , (A_{L}, b_{L}) \bigr)$$

be a tuple of matrix–vector pairs where $A_{l}\in \mathbb{R}^{N_{l}\times N_{l-1}}$, $b_{l}\in \mathbb{R}^{N_{l}}$ and $N_{0} = d$, $N_{L} = m$. Every matrix–vector pair $(A_{l}, b_{l})$ induces an affine linear map $T_{l}\colon \mathbb{R}^{N_{l-1}} \to \mathbb{R}^{N_{l}}$. The *neural network function with parameters* *θ* and with respect to some *activation function* $\rho \colon \mathbb{R}\to \mathbb{R}$ is the function

$$ u^{\rho}_{\theta}\colon \mathbb{R}^{d}\to \mathbb{R}^{m}, \qquad x \mapsto T_{L}\bigl(\rho \bigl(T_{L-1} \bigl(\rho \bigl(\cdots \rho \bigl(T_{1}(x)\bigr)\bigr)\bigr)\bigr) \bigr).$$

The set of all neural network functions of a certain architecture is given by $\{ u^{\rho}_{\theta}\mid \theta \in \Theta \}$, where Θ collects all parameters of the above form with respect to fixed natural numbers $d,m,L,N_{0},\dots ,N_{L}$. If we have $f=u_{\theta}^{\rho}$ for some $\theta \in \Theta $ we say the function *f* can be *realized* by the neural network $\mathcal {F}^{\rho}_{\Theta}$. Note that we often drop the superscript *ρ* if it is clear from the context.

A particular activation function often used in practice and relevant for our results is the *rectified linear unit* or *ReLU activation function*, which is defined via $x\mapsto \max \{ 0, x \}$. Arora et al. [2] showed that the class of ReLU networks coincides with the class of continuous and piecewise linear functions. In particular, they are weakly differentiable. Since piecewise linear functions are dense in $H^{1}_{0}(\Omega )$, we obtain the following universal approximation result which we prove in detail in the appendix.

#### Theorem 2

(Universal approximation with zero boundary values)

*Consider an open set* $\Omega \subseteq \mathbb{R}^{d}$ *and fix a function* $u\in W^{1,p}_{0}(\Omega )$ *with* $p\in [1,\infty )$. *Then for all* $\varepsilon >0$ *there exists* $u_{\varepsilon}\in W^{1,p}_{0}(\Omega )$ *that can be realized by an ReLU network of depth* $\lceil \log _{2}(d+1)\rceil +1$ *such that*

$$ \lVert u - u_{\varepsilon } \rVert _{W^{1,p}(\Omega )} \le \varepsilon .$$

To the best of our knowledge, this is the only available universal approximation result where the approximating neural network functions are guaranteed to have zero boundary values. This relies on the special properties of the ReLU activation function and it is unclear for which classes of activation functions universal approximation with zero boundary values hold.

### Gamma convergence

We recall the definition of Γ-convergence with respect to the weak topology of reflexive Banach spaces. For further reading, we point the reader towards Dal Maso [9].

#### Definition 3

(Γ-convergence)

Let *X* be a reflexive Banach space as well as $F_{n}, F\colon X\to (-\infty , \infty ]$. Then $(F_{n})_{n\in \mathbb{N}}$ is said to be Γ*-convergent* to *F* if the following two properties are satisfied:

(i) (*Liminf inequality*) For every $x\in X$ and $(x_{n})_{n\in \mathbb{N}}$ with $x_{n}\rightharpoonup x$, we have
  $$ F(x) \le \liminf_{n\to \infty} F_{n}(x_{n}).$$
(ii) (*Recovery sequence*) For every $x\in X$, there is $(x_{n})_{n\in \mathbb{N}}$ with $x_{n}\rightharpoonup x$ such that
  $$ F(x) = \lim_{n\to \infty} F_{n}(x_{n}).$$

The sequence $(F_{n})_{n\in \mathbb{N}}$ is called *equicoercive* if the set

$$ \bigcup_{n\in \mathbb{N}} \bigl\{ x\in X \mid F_{n}(x) \le r \bigr\} $$

is bounded in *X* (or equivalently, relatively compact with respect to the weak topology) for all $r\in \mathbb{R}$. We say that a sequence $(x_{n})_{n\in \mathbb{N}}$ are *quasiminimizers* of the functionals $(F_{n})_{n\in \mathbb{N}}$ if we have

$$ F_{n}(x_{n}) \le \inf_{x\in X} F_{n}(x) + \delta _{n}, $$

where $\delta _{n}\to 0$.

We need the following property of Γ-convergent sequences. We want to emphasize the fact that there are no requirements regarding the continuity of any of the functionals and that the functionals $(F_{n})_{n\in \mathbb{N}}$ are not assumed to admit minimizers.

#### Theorem 4

(Convergence of quasiminimizers)

*Let* *X* *be a reflexive Banach space and* $(F_{n})_{n\in \mathbb{N}}$ *be an equicoercive sequence of functionals that* Γ-*converges to* *F*. *Then*, *any sequence* $(x_{n})_{n\in \mathbb{N}}$ *of quasiminimizers of* $(F_{n})_{n\in \mathbb{N}}$ *is relatively compact with respect to the weak topology of* *X* *and every weak accumulation point of* $(x_{n})_{n\in \mathbb{N}}$ *is a global minimizer of* *F*. *Consequently*, *if* *F* *possesses a unique minimizer* *x*, *then* $(x_{n})_{n\in \mathbb{N}}$ *converges weakly to* *x*.

## Abstract Γ-convergence result for the deep Ritz method

For the abstract results, we work with an abstract energy $E\colon X\to \mathbb{R}$, instead of an integral functional of the form (1). This reduces technicalities in the proofs and separates abstract functional-analytic considerations from applications.

### Setting 5

*Let* $(X, \lVert \cdot \rVert _{X})$ *and* $(B, \lVert \cdot \rVert _{B})$ *be reflexive Banach spaces and* $\gamma \in \mathcal{L}(X,B)$ *be a continuous linear map*. *We set* $X_{0}$ *to be the kernel of* *γ*, *i*.*e*., $X_{0} = \gamma ^{-1}( \{0\})$. *Let* $\rho \colon \mathbb{R} \to \mathbb{R}$ *be some activation function and denote by* $(\Theta _{n})_{n\in \mathbb{N}}$ *a sequence of neural network parameters*. *We assume that any function represented by such a neural network is a member of* *X* *and define*

6 $$ A_{n} := \{ x_{\theta }\mid \theta \in \Theta _{n} \} \subseteq X. $$

*Here*, $x_{\theta}$ *denotes the function represented by the neural network with the parameters* *θ*. *Let* $E\colon X\to (-\infty ,\infty ]$ *be a functional and* $(\lambda _{n})_{n\in \mathbb{N}}$ *a sequence of real numbers with* $\lambda _{n}\to \infty $. *Furthermore*, *let* $p\in (1,\infty )$ *and* $f\in X^{*}$ *be fixed and define the functional* $F^{f}_{n}\colon X\to (-\infty ,\infty ]$ *by*

$$\begin{aligned} F^{f}_{n}(x) = \textstyle\begin{cases} E(x) + \lambda _{n}\lVert \gamma (x)\rVert ^{p}_{B} - f(x) &\textit{for }x\in A_{n}, \\ \infty &\textit{otherwise}, \end{cases}\displaystyle \end{aligned}$$

*as well as* $F^{f}\colon X\to (-\infty ,\infty ]$ *by*

$$\begin{aligned} F^{f}(x) = \textstyle\begin{cases} E(x) - f(x) &\textit{for } x\in X_{0}, \\ \infty &\textit{otherwise}. \end{cases}\displaystyle \end{aligned}$$

*Then assume the following holds*:

1. *For every* $x\in X_{0}$, *there is* $x_{n}\in A_{n}$ *such that* $x_{n}\to x$ *and* $\lambda _{n}\lVert \gamma (x_{n}) \rVert ^{p}_{B} \to 0$ *for* $n\to \infty $.
2. *The functional* *E* *is bounded from below*, *weakly lower semicontinuous with respect to the weak topology of* $(X, \lVert \cdot \rVert _{X})$ *and continuous with respect to the norm topology of* $(X, \lVert \cdot \rVert _{X})$.
3. *The sequence* $(F^{f}_{n})_{n\in \mathbb{N}}$ *is equicoercive with respect to the norm* $\lVert \cdot \rVert _{X}$.

### Remark 6

We discuss Assumptions (A1) to (A3) in view of their applicability to concrete problems.

(i) In applications, $(X,\lVert \cdot \rVert _{X})$ will usually be a Sobolev space with its natural norm, the space *B* contains boundary values of functions in *X* and the operator *γ* is a boundary value operator, e.g., the trace map. However, if the energy *E* is coercive on all of *X*, i.e., without adding boundary terms to it, we might choose $\gamma = 0$ and obtain $X_{0} = X$. This is the case for non-essential boundary value problems.
(ii) Assumption (A1) compensates that, in general, we cannot penalize with arbitrary strength. However, if we can approximate any member of $X_{0}$ by a sequence $x_{\theta _{n}}\in A_{n}\cap X_{0}$ then any divergent sequence $(\lambda _{n})_{n\in \mathbb{N}}$ can be chosen. This is, for example, the case for the ReLU activation function and the space $X_{0}=H^{1}_{0}(\Omega )$. More precisely, we can choose $A_{n}$ to be the class of functions expressed by a (fully connected) ReLU network of depth $\lceil \log _{2}(d+1)\rceil +1$ and width *n*, see Theorem 2.

### Theorem 7

(Γ-convergence)

*Assume we are in Setting* 5. *Then the sequence* $(F^{f}_{n})_{n\in \mathbb{N}}$ *of functionals* Γ-*converges towards* $F^{f}$. *In particular*, *if* $(\delta _{n})_{n\in \mathbb{N}}$ *is a sequence of nonnegative real numbers converging to zero*, *any sequence of* $\delta _{n}$-*quasiminimizers of* $F_{n}^{f}$ *is bounded and all its weak accumulation points are minimizers of* $F^{f}$. *If*, *additionally*, $F^{f}$ *possesses a unique minimizer* $x^{f} \in X_{0}$, *any sequence of* $\delta _{n}$-*quasiminimizers converges to* $x^{f}$ *in the weak topology of* *X*.

### Proof

We begin with the limes inferior inequality. Let $x_{n}\rightharpoonup x$ in *X* and assume that $x\notin X_{0}$. Then $f(x_{n})$ converges to $f(x)$ as real numbers and $\gamma (x_{n})$ converges weakly to $\gamma (x)\neq 0$ in *B*. Combining this with the weak lower semicontinuity of $\rVert \cdot \lVert ^{p}_{B} $, we get, using the boundedness from below, that

$$ \liminf_{n\to \infty}F_{n}^{f}(x_{n}) \geq \inf_{x\in X} E(x) + \liminf_{n\to \infty}\lambda _{n}\bigl\lVert \gamma (x_{n})\bigr\rVert ^{p}_{B} - \lim_{n\to \infty}f(x_{n}) = \infty . $$

Now let $x\in X_{0}$. Then by the weak lower semicontinuity of *E*, we find

$$ \liminf_{n\to \infty}F_{n}^{f}(x_{n}) \geq \liminf_{n\to \infty}E(x_{n}) - f(x) \geq E(x) -f(x) = F^{f}(x). $$

Now let us have a look at the construction of the recovery sequence. For $x\notin X_{0}$, we can choose the constant sequence and estimate

$$ F^{f}_{n}(x_{n}) \geq E(x) + \lambda _{n}\bigl\lVert \gamma (x)\bigr\rVert _{B}^{p} - f(x). $$

Hence we find that $F_{n}^{f_{n}}(x)\to \infty = F^{f}(x)$. If $x\in X_{0}$, we approximate it with a sequence $(x_{n})\subseteq X$, according to Assumption (A1), such that $x_{n}\in A_{n}$ and $x_{n}\to x$ in $\lVert \cdot \rVert _{X}$ and $\lambda _{n}\lVert \gamma (x_{n}) \rVert _{B}^{p} \to 0$. It follows that

$$ F_{n}^{f}(x_{n}) = E(x_{n}) + \lambda _{n}\lVert x_{n} \rVert _{B}^{p} -f(x_{n}) \to E(x)-f(x) = F^{f}(x). $$

 □

A sufficient criterion for equicoercivity of the sequence $(F^{f}_{n})_{n\in \mathbb{N}}$ from Assumption (A3) in terms of the functional *E* is given by the following lemma.

### Lemma 8

(Criterion for equicoercivity)

*Assume we are in Setting* 5. *If there is a constant* $c>0$ *such that it holds for all* $x\in X$ *that*

$$ E(x) + \bigl\lVert \gamma (x) \bigr\rVert _{B}^{p} \geq c \cdot \bigl( \lVert x \rVert _{X}^{p} - \lVert x \rVert _{X} - 1 \bigr), $$

*then the sequence* $(F^{f}_{n})_{n\in \mathbb{N}}$ *is equicoercive*.

### Proof

It suffices to show that the sequence

$$ G_{n}^{f}\colon X\to \mathbb{R}\quad \text{with } G^{f}_{n}(x) = E(x) + \lambda _{n}\bigl\lVert \gamma (x)\bigr\rVert _{B}^{p} - f(x) $$

is equicoercive, as $G^{f}_{n}\leq F_{n}^{f}$. So let $r\in \mathbb{R}$ be given and assume that $r\geq G^{f}_{n}(x)$. We estimate, assuming without loss of generality that $\lambda _{n} \geq 1$,

$$\begin{aligned} r &\geq E(x) + \lambda _{n}\bigl\lVert \gamma (x)\bigr\rVert _{B}^{p} - f(x) \\ &\geq c \cdot \bigl(\lVert x \rVert _{X}^{p} - \lVert x \rVert _{X} - 1 \bigr) - \lVert f\rVert _{X^{*}}\cdot \lVert x \rVert _{X} \\ &\geq \tilde{c} \cdot \bigl(\lVert x \rVert _{X}^{p} - \lVert x \rVert _{X} - 1 \bigr). \end{aligned}$$

As $p> 1$, a scaled version of Young’s inequality clearly implies a bound on the set

$$ \bigcup_{n\in \mathbb{N}} \bigl\{ x\in X\mid G_{n}^{f}(x) \leq r \bigr\} $$

and hence the sequence $(F^{f}_{n})_{n\in \mathbb{N}}$ is seen to be equicoercive. □

## Abstract uniform convergence result for the deep Ritz method

In this section we present an extension of Setting 5 that allows proving uniform convergence results over certain bounded families of right-hand sides.

### Setting 9

*Assume we are in Setting* 5. *Furthermore*, *let there be an additional norm* $\lvert \cdot \rvert $ *on* *X* *such that the dual space* $(X,\lvert \cdot \rvert )^{*}$ *is reflexive*. *However*, *we do not require* $(X,\lvert \cdot \rvert )$ *to be complete*. *Then*, *let the following assumptions hold*:

(A4) *The identity* $\operatorname{Id}\colon (X, \lVert \cdot \rVert _{X}) \to (X,\lvert \cdot \rvert )$ *is completely continuous*, *i*.*e*., *maps weakly convergent sequences to strongly convergent ones*.
(A5) *For every* $f\in X^{*}$, *there is a unique minimizer* $x_{f}\in X_{0}$ *of* $F^{f}$ *and the solution map*
  $$ S\colon X_{0}^{*}\to X_{0}\quad \textit{with } f \mapsto x^{f} $$
  *is demicontinuous*, *i*.*e*., *maps strongly convergent sequences to weakly convergent ones*.

### Remark 10

As mentioned earlier, $(X, \lVert \cdot \rVert _{X})$ is usually a Sobolev space with its natural norm. The norm $\lvert \cdot \rvert $ may then chosen to be an $L^{p}(\Omega )$ or $W^{s,p}(\Omega )$ norm, where *s* is strictly smaller than the differentiability order of *X*. In this case, Rellich’s compactness theorem provides Assumption (A4).

### Lemma 11

(Compactness)

*Assume we are in Setting* 9. *Then the solution operator* $S\colon (X,\lvert \cdot \rvert )^{*}\to (X_{0},\lvert \cdot \rvert )$ *is completely continuous*, *i*.*e*., *maps weakly convergent sequences to strongly convergent ones*.

### Proof

We begin by clarifying what we mean by *S* being defined on $(X,\lvert \cdot \rvert )^{*}$. Denote by *i* the inclusion map $i\colon X_{0}\to X$ and consider

$$\begin{aligned} \bigl(X,\lvert \cdot \rvert \bigr)^{*} \stackrel{\operatorname{Id}^{*}}{\longrightarrow} \bigl(X, \lVert \cdot \rVert _{X} \bigr)^{*} \stackrel{i^{*}}{\longrightarrow}\bigl(X_{0}, \lVert \cdot \rVert _{X}\bigr)^{*} \stackrel{S}{\longrightarrow} \bigl(X_{0}, \lVert \cdot \rVert _{X}\bigr) \stackrel{\operatorname{Id}}{\longrightarrow} \bigl(X_{0}, \lvert \cdot \rvert \bigr). \end{aligned}$$

By abusing notation, always when we refer to *S* as defined on $(X,\lvert \cdot \rvert )^{*}$ we mean the above composition, i.e., $\operatorname{Id}\circ S\circ i^{*}\circ \operatorname{Id}^{*}$. Having explained this, it is clear that it suffices to show that Id^∗^ maps weakly convergent sequences to strongly convergent ones since $i^{*}$ is continuous, *S* demicontinuous, and Id strongly continuous. This, however, is a consequence of Schauder’s theorem, see, for instance, Alt [1], which states that a linear map $L\in \mathcal{L}(X,Y)$ between Banach spaces is compact if and only if $L^{*}\in \mathcal{L}(Y^{*},X^{*})$ is. Here, compact means that *L* maps bounded sets to relatively compact ones. Let $X_{c}$ denote the completion of $(X,\lvert \cdot \rvert )$. Then, using the reflexivity of $(X, \lVert \cdot \rVert _{X})$ it is easily seen that $\operatorname{Id}\colon (X, \lVert \cdot \rVert _{X}) \to X_{c}$ is compact. Finally, using that $(X,\lvert \cdot \rvert )^{*} = X_{c}^{*}$ the desired compactness of Id^∗^ is established. □

The following theorem is the main result of this section. It shows that the convergence of the Deep Ritz method is uniform on bounded sets in the space $(X, \lvert \cdot \rvert )^{*}$. The proof of the uniformity follows an idea from Cherednichenko et al. [7], where in a different setting a compactness result was used to amplify pointwise convergence to uniform convergence across bounded sets, compare to Theorem 4.1 and Corollary 4.2 in Cherednichenko et al. [7].

### Theorem 12

(Uniform convergence of the Deep Ritz Method)

*Assume that we are in Setting* 9*and let* $\delta _{n}\searrow 0$ *be a sequence of real numbers*. *For* $f\in X^{*}$, *we set*

$$ S_{n}(f) := \Bigl\{ x\in X \bigm\lvert F^{f}_{n}(x) \leq \inf_{z\in X} F_{n}^{f}(z) + \delta _{n} \Bigr\} , $$

*which is the approximate solution set corresponding to* *f* *and* $\delta _{n}$. *Furthermore*, *denote the unique minimizer of* $F^{f}$ *in* $X_{0}$ *by* $x^{f}$ *and fix* $R>0$. *Then we have*

$$ \sup \bigl\{ \bigl\lvert x^{f}_{n}-x^{f}\bigr\rvert \mid x_{n}^{f} \in S_{n}(f), \lVert f \rVert _{(X, \lvert \cdot \rvert )^{*}} \leq R \bigr\} \to 0 \quad \textit{for } n\to \infty . $$

In the definition of this supremum, *f* is measured in the norm of the space $(X, \lvert \cdot \rvert )^{*}$. This means that $f:(X, \lvert \cdot \rvert )\to \mathbb{R}$ is continuous, which is a more restrictive requirement than the continuity with respect to $\lVert \cdot \rVert _{X}$. Also the computation of this norm takes place in the unit ball of $(X, \lvert \cdot \rvert )$, i.e.,

$$ \lVert f \rVert _{(X, \lvert \cdot \rvert )^{*}} = \sup_{ \lvert x \rvert \leq 1}f(x). $$

Before we prove Theorem 12, we need a Γ-convergence result similar to Theorem 7. The only difference is that now also the right-hand side may vary along the sequence.

### Proposition 13

*Assume that we are in Setting* 9, *however*, *we do not need Assumption* (A5) *for this result*. *Let* $f_{n},f\in (X,\lvert \cdot \rvert )^{*}$ *be such that* $f_{n}\rightharpoonup f$ *in the weak topology of the reflexive space* $(X,\lvert \cdot \rvert )^{*}$. *Then the sequence* $(F_{n}^{f_{n}})_{n\in \mathbb{N}}$ *of functionals* Γ-*converges to* $F^{f}$ *in the weak topology of* $(X, \lVert \cdot \rVert _{X})$. *Furthermore*, *the sequence* $(F_{n}^{f_{n}})_{n\in \mathbb{N}}$ *is equicoercive*.

### Proof

The proof is almost identical to that of Theorem 7 but, since it is brief, we include it for the reader’s convenience. We begin with the limes inferior inequality. Let $x_{n}\rightharpoonup x$ in *X* and $x\notin X_{0}$. Then $x_{n}\to x$ with respect to $\lvert \cdot \rvert $ which implies that $f_{n}(x_{n})$ converges to $f(x)$. Using that $\gamma (x_{n})\rightharpoonup \gamma (x) $ in *B*, combined with the weak lower semicontinuity of $\rVert \cdot \lVert ^{p}_{B} $, we get

$$ \liminf_{n\to \infty}F_{n}^{f_{n}}(x_{n}) \geq \inf_{x\in X} E(x) + \liminf_{n\to \infty}\lambda _{n}\bigl\lVert \gamma (x_{n})\bigr\rVert ^{p}_{B} - \lim_{n\to \infty}f_{n}(x_{n}) = \infty . $$

Now let $x\in X_{0}$. Then by the weak lower semicontinuity of *E*, we find

$$ \liminf_{n\to \infty}F_{n}^{f_{n}}(x_{n}) \geq \liminf_{n\to \infty}E(x_{n}) - f(x) \geq E(x) -f(x) = F^{f}(x). $$

Now let us have a look at the construction of the recovery sequence. For $x\notin X_{0}$, we can choose the constant sequence and estimate

$$ F^{f_{n}}_{n}(x) \geq \inf_{x\in X}E(x) + \lambda _{n}\bigl\lVert \gamma (x) \bigr\rVert _{B}^{p} - \lVert f_{n}\rVert _{(X,\lvert \cdot \rvert )'} \cdot \lvert x\rvert . $$

As $\lVert f_{n}\rVert _{(X,\lvert \cdot \rvert )^{*}}$ is bounded we find $F_{n}^{f_{n}}(x)\to \infty = F^{f}(x)$. If $x\in X_{0}$, we approximate it with a sequence $(x_{n})\subseteq X$, according to Assumption (A1), such that $x_{n}\in A_{n}$ and $x_{n}\to x$ in $\lVert \cdot \rVert _{X}$ and $\lambda _{n}\lVert \gamma (x_{n}) \rVert _{B}^{p} \to 0$. It follows that

$$ F_{n}^{f_{n}}(x_{n}) = E(x_{n}) + \lambda _{n}\lVert x_{n} \rVert _{B}^{p} -f_{n}(x_{n}) \to E(x)-f(x) = F^{f}(x). $$

The equicoercivity was already assumed in (A3) so it does not need to be shown. □

### Proof of Theorem 12

We can choose $(f_{n})\subseteq (X,\lvert \cdot \rvert )^{*}$ and $\lVert f_{n}\rVert _{(X,\lvert \cdot \rvert )^{*}}\leq R$ and $x_{n}^{f_{n}}\in S_{n}(f_{n})$ such that

$$ \sup_{ \begin{subarray}{c} \lVert f\rVert _{(X, \lvert \cdot \rvert )^{*}}\leq R x^{f}_{n} \in S_{n}(f) \end{subarray} } \bigl\lvert x^{f}_{n}-x^{f} \bigr\rvert \leq \bigl\lvert x_{n}^{f_{n}}-x^{f_{n}} \bigr\rvert + \frac{1}{n}. $$

Now it suffices to show that $\lvert x_{n}^{f_{n}} - x^{f_{n}}\rvert $ converges to zero. Since $(f_{n})_{n\in \mathbb{N}}$ is bounded in $(X,\lvert \cdot \rvert )^{*}$ and this space is reflexive, we can, without loss of generality, assume that $f_{n}\rightharpoonup f$ in $(X,\lvert \cdot \rvert )^{*}$. This implies by Lemma 11 that $x^{f_{n}}\to x^{f}$ in $(X,\lvert \cdot \rvert )$. The Γ-convergence result of the previous proposition yields $x_{n}^{f_{n}}\rightharpoonup x^{f}$ in *X* and hence $x_{n}^{f_{n}}\to x^{f}$ with respect to $\lvert \cdot \rvert $ which concludes the proof. □

## Examples

We discuss different concrete examples that allow the application of our abstract results and focus on nonlinear problems. In particular, we consider a phase field model illustrating the basic Γ-convergence result of Sect. 3 and the *p*-Laplacian as an example for the uniform results of Sect. 4.

### General practical considerations

In practice, when solving the optimization problem (4), in order to obtain an approximate solution of the variational problem (2) there are a lot of choices to make. We give an overview over some of them here and report our specific choices in the individual examples.

#### Optimization

One can use almost any kind of optimization algorithm for the approximation solution of the optimization problem (4), where gradient type algorithms and quasi-Newton methods are the most common choice. We use a combination of the Adam optimizer and L-BFGS. The former is a version of stochastic gradient descent with with adaptive moment estimation, see Kingma and Ba [19] and the latter is a quasi-Newton method, see Liu and Nocedal [22]. Typically, the optimization process begins with applying Adam until convergence slows down and the fast local convergence properties of Newton methods can be exploited through the application of the L-BFGS optimizer.

#### Quadrature

In practice, one does not have access to the true gradient of the objective function. Hence, one usually uses estimates for the gradient as update directions. For example, E and Yu [13] used an online SGD estimator, i.e., a Monte Carlo approximation of the integral with fixed sample size. However, one can use any quadrature rule for the evaluation of the integrals in order to obtain approximations of the true gradient. We used a uniform grid for the discretization of the integral, i.e., the integral is approximated by the sum of the functions values at the grid points divided by the number of grid points. On the boundary of the domains, equispaced integration points are used and the quadrature rule is analogue to the one previously described.

We choose the number of integration points such that no further improved accuracy of the method is observed when increasing their amount. As this was computationally tractable without problems, no more elaborate integration routines were deemed necessary.

#### Activation functions

The only requirements on the activation function present in Setting 5 are that the associated neural network functions belong to the considered Banach space $A_{n}\subseteq X$ as well as that Condition (A1) holds. The first one is usually of no concern as in practice *X* is often a space of given smoothness and hence for sufficently smooth activations $A_{n}\subseteq X$ is satisfied. Note that this is in particular the case $X= H^{1}(\Omega )$ and for the ReLU activation function. Further, in order to Condition (A1) to hold, it is necessary that the neural network type ansatz classes $(A_{n})$ have the universal approximation property in $X_{0}$, i.e., that $X_{0}\subseteq \overline{\bigcup_{n\in \mathbb{N}} A_{n}}$. Note that this is the case for shallow^1^ networks of increasing width and $X=H^{k}(\Omega )$ as long as the activation function is *k* times continuously differentiable and nonpolynomial (Pinkus [27]).

#### Penalization strength

Condition (A1) couples the penalization strength to the norm of the (generalized) boundary values required for approximation of a general element $x\in X$. Consider, for example, the case that for any $x\in X_{0}$ there are $x_{n}\in X_{n}$ such that $x_{n}\to x$ and $\lVert \gamma (x_{n})\rVert _{B} \le c(x) \delta _{n}$ for some $\delta _{n}\to 0$. Then any choice of penalization strengths $\lambda _{n}\to \infty $ with $\lambda _{n}\delta _{n}\to 0$ satisfies Condition (A1). Let us first consider the case with inessential boundary values, which corresponds to $\gamma \equiv 0$ in the notation of Setting 5. Then, as argued above smooth and nonpolynomial activations together with arbitrarily strong penalization strengths are allowed.

Let us consider the case $X=H^{1}(\Omega )$ and $\gamma =\operatorname{tr}$ and $B=L^{2}(\Omega )$. For the ReLU activation function, Theorem 2 guarantees the existence of the $u_{n}\to u$ with $\lVert u_{n}\rVert _{L^{2}(\partial \Omega )}=0$, which allows for arbitrarily strong penalization. For other activation functions, which do not possess the universal approximation property with exact (generalized) zero boundary values, the proof of (A1) is more delicate and has to be established in specific cases.

### A phase field model

Let $\varepsilon > 0$ be fixed, $\Omega \subseteq \mathbb{R}^{d}$ a bounded Lipschitz domain and consider the following energy:

$$ E\colon H^{1}(\Omega ) \cap L^{4}(\Omega ) \to [0,\infty ), \qquad E(u) = \frac{\varepsilon}{2} \int _{\Omega } \vert \nabla u \vert ^{2}\,\mathrm{d}x + \frac{1}{\varepsilon} \int _{\Omega }W(u)\,\mathrm{d}x, $$

where $W\colon \mathbb{R}\to \mathbb{R}$ is the nonlinear function given by

$$ W(u) = \frac{1}{4}u^{2}(u-1)^{2} = \frac{1}{4} u^{4} - \frac{1}{2} u^{3} + \frac{1}{4} u^{2}. $$

The functional *E* constitutes a way to approximately describe phase separation and the parameter *ε* encodes the length-scale of the phase transition, see Cahn and Hilliard [6]. We describe now how the Setting 5 is applicable to fully connected neural network ansatz functions with tanh activation function. For the Banach spaces in Setting 5, we choose

$$\begin{aligned}& X = H^{1}(\Omega )\cap L^{4}(\Omega ), \qquad \lVert \cdot \rVert _{X} = \lVert \cdot \rVert _{H^{1}( \Omega )} + \lVert \cdot \rVert _{L^{4}(\Omega )}. \end{aligned}$$

We choose $\gamma \equiv 0$, hence the choice of the space *B* and its norm is irrelevant. The choice of $\gamma \equiv 0$ corresponds to the case of homogeneous Neumann boundary conditions. The space *X* is reflexive as it is an intersection of reflexive spaces. We define

$$ A_{n}:= \{ u_{\theta }\mid \theta \in \Theta _{n} \} \subseteq H^{1}(\Omega )\cap L^{4}(\Omega ), $$

where $\Theta _{n}$ implicitly encodes that we use scalar valued neural networks with input dimension *d* and arbitrary fixed depth larger or equal to two. The width of all layers (except the input and output) is set to *n*. With $\gamma \equiv 0$ and this definition of $(A_{n})_{n\in \mathbb{N}}$ the requirements of Assumption (A1) are satisfied, as can be seen by well known universal approximation results, we refer to Pinkus [27].

To proceed, the continuity of *E* with respect to $\lVert \cdot \rVert _{X}$ is clear, hence we turn to the weak lower semicontinuity. To this end, we write *E* in the following form:

$$\begin{aligned} E(u) = \underbrace{\frac{\varepsilon}{2} \int _{\Omega } \vert \nabla u \vert ^{2}\,\mathrm{d}x + \frac{1}{4\varepsilon} \int _{\Omega }u^{4}\,\mathrm{d}x}_{ := E_{1}(u)} + \underbrace{\frac{1}{\varepsilon} \int _{\Omega }\frac{1}{4} u^{2} - \frac{1}{2} u^{3}\,\mathrm{d}x }_{ := E_{2}(u)} \end{aligned}$$

and treat $E_{1}$ and $E_{2}$ separately. The term $E_{1}$ is continuous with respect to $\lVert \cdot \rVert _{X}$ and convex, hence weakly lower semicontinuous. To treat $E_{2}$, note that we have the compact embedding

$$ H^{1}(\Omega )\cap L^{4}(\Omega ) \hookrightarrow \hookrightarrow L^{3}( \Omega ). $$

This implies that a sequence that converges weakly in $H^{1}(\Omega )\cap L^{4}(\Omega )$ converges strongly in $L^{3}(\Omega )$ and consequently shows that the term $E_{2}$ is continuous with respect to weak convergence in *X*. Finally, for fixed $f\in X^{*}$, we need to show that the sequence $(F^{f}_{n})_{n\in \mathbb{N}}$ defined in (6) is equicoercive with respect to $\lVert \cdot \rVert _{X}$. To this end, it suffices to show that the functional

$$ G^{f}\colon X\to \mathbb{R}, \qquad G^{f}(u) = \frac{\varepsilon}{2} \int _{\Omega } \vert \nabla u \vert ^{2}\,\mathrm{d}x + \frac{1}{\varepsilon} \int _{ \Omega }W(u)\,\mathrm{d}x - f(u) $$

is coercive as it holds $F^{f}_{n} \geq G^{f}$. Let $r\in \mathbb{R}$ be fixed and consider all $u\in X$ with $G^{f}(u) \geq r$. Then we estimate

$$\begin{aligned} r &\geq G^{f}(u) \\ &\geq \frac{\varepsilon}{2} \int _{\Omega } \vert \nabla u \vert ^{2} \,\mathrm{d}x + \frac{1}{\varepsilon} \int _{\Omega }W(u)\,\mathrm{d}x - f(u) \\ &\geq c \lVert u \rVert ^{2}_{H^{1}(\Omega )} - \lVert f \rVert _{X^{*}} \bigl( \lVert u \rVert _{H^{1}(\Omega )} + \lVert u \rVert _{L^{4}(\Omega )} \bigr) + \frac{1}{4\varepsilon} \lVert u \rVert ^{4}_{L^{4}(\Omega )} - \frac{1}{3\varepsilon} \lVert u \rVert ^{3}_{L^{3}(\Omega )} \\ &\geq c \lVert u \rVert ^{2}_{H^{1}(\Omega )} - \lVert f \rVert _{X^{*}} \lVert u \rVert _{H^{1}(\Omega )} + \frac{1}{4\varepsilon} \lVert u \rVert ^{4}_{L^{4}(\Omega )} - \frac{ \vert \Omega \vert ^{1/4}}{3\varepsilon} \lVert u \rVert ^{3/4}_{L^{4}( \Omega )} - \lVert f \rVert _{X^{*}}\lVert u \rVert _{L^{4}(\Omega )}, \end{aligned}$$

where we used the estimate

$$ \lVert u \rVert _{L^{3}(\Omega )}^{3} \leq \vert \Omega \vert ^{1/4} \lVert u \rVert _{L^{4}(\Omega )}^{3/4} $$

due to Hölder’s inequality. This clearly implies a bound on the set

$$ \bigcup_{n\in \mathbb{N}} \bigl\{ u\in H^{1}(\Omega ) \cap L^{4}( \Omega ) \mid G^{f}(u) \leq r \bigr\} $$

and hence $(F^{f}_{n})_{n\in \mathbb{N}}$ is equicoercive.

#### Description of the experiment

**Figure 1:**
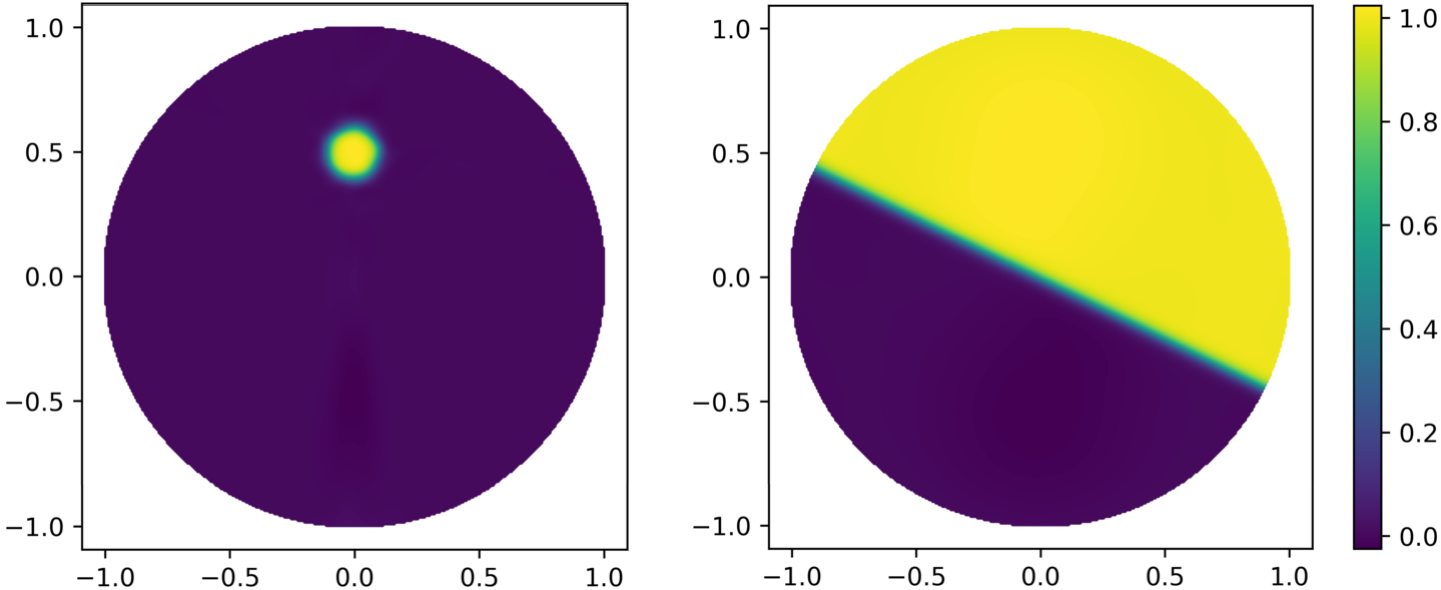
Exemplary numerical realization of the Deep Ritz Method for a Cahn–Hilliard functional with right-hand sides given through $f=\chi _{B_{r}(0,-1/2)} - \chi _{B_{r}(0,1/2)}$ with $r=0.1$ for the left plot and $r=0.4$ for the right plot. The value of *ε* is set to 0.01. We used zero Neumann boundary conditions and fully connected feed-forward networks with three hidden layers of width 16 and tanh activation. The number of trainable parameters is 609

Figure 1 shows two exemplary numerical realizations of the Deep Ritz Method with the unit disk $\Omega =B_{1}(0)$ as a domain and with right-hand sides

$$ f_{i} = \chi _{B_{r_{i}}(0,-1/2)} - \chi _{B_{r_{i}}(0,1/2)} $$

for $r_{1}=0.1$ and $r_{2}=0.4$ corresponding to the left and right picture, respectively. Further, we considered $\varepsilon =0.01$ and used a fully connected network with tanh activation and three hidden layers of width 16. Note that by Theorem 2 ReLU networks of depth $\lceil \log _{2}(2+1)\rceil +1=3$ satisfy the universal approximation property with exact zero boundary values. Hence, the number of trainable parameters is 609 in this case. As we were solving a homogeneous Neumann boundary value problem, no penalization was needed. For the discretization of the integral over the unit disk $B_{1}(0)$, we used an evenly spaced grid and gave equal weights in the numerical approximation of the integrals to the function values at every grid point. For the optimization of the networks parameters, we used Adam with full batch size until the optimization slowed down and then used L-BFGS in order to exploit the fast local convergence properties of quasi-Newton methods. Note that in the case of $f_{1}$, a phase transition around the ball $B_{r_{1}}(0,1/2)$ is energetically more favorable than the configuration in the right figure, where the radius $r_{2}$ is much larger.

#### Remark 14

(Stability under compact perturbations)

With a similar – even simpler – approach, we may also show that energies of the form

$$ \hat{E}(u) = E(u) + F(u) $$

fall in the Setting 5 provided *E* does and *F* is bounded from below and continuous with respect to weak convergence in *X*. Note also that in the space dimension $d = 2$ this includes the above example, however, the slightly more involved proof presented here works independently of the space dimension *d*.

### The *p*-Laplacian

As an example for the uniform convergence of the Deep Ritz method, we discuss the *p*-Laplacian. To this end, consider the *p*-Dirichlet energy for $p\in (1,\infty )$ given by

$$\begin{aligned} E\colon W^{1,p}(\Omega )\to \mathbb{R} ,\qquad u \mapsto \frac{1}{p} \int _{ \Omega} \vert \nabla u \vert ^{p} \,\mathrm{d}x. \end{aligned}$$

Note that for $p\neq 2$ the associated Euler–Lagrange equation – the *p*-Laplace equation – is nonlinear. In strong formulation it is given by

$$\begin{aligned} &{-}\operatorname{div}\bigl(\lvert \nabla u\rvert ^{p-2}\nabla u\bigr) = f \quad \text{in }\Omega , \\ &u = 0 \quad \text{on }\partial \Omega , \end{aligned}$$

see, for example, Struwe [31] or Růžička [29]. Choosing the ReLU activation function, the abstract setting is applicable as we will describe now. For the Banach spaces, we choose

$$ X = W^{1,p}(\Omega ), \qquad B = L^{p}(\partial \Omega ), \qquad \lvert u\rvert = \lVert u \rVert _{L^{p}(\Omega )},$$

where the norms $\lVert \cdot \rVert _{X}$ and $\lVert \cdot \rVert _{B}$ are chosen to be the natural ones. Clearly, $W^{1,p}(\Omega )$ endowed with the norm $\lVert \cdot \rVert _{W^{1,p}(\Omega )}$ is reflexive by our assumption $p\in (1,\infty )$. Note that

$$ \bigl(W^{1,p}(\Omega ), \lVert \cdot \rVert _{L^{p}( \Omega )} \bigr)^{*} = L^{p}(\Omega )^{*} \cong L^{p^{\prime}}( \Omega ), $$

which is also reflexive. We set $\gamma = \operatorname{tr}$, i.e.,

$$\begin{aligned} \operatorname{tr}\colon W^{1,p}(\Omega ) & \to L^{p}(\partial \Omega ) \quad \text{with } u\mapsto u|_{\partial \Omega} \end{aligned}$$

We use the same ansatz sets $(A_{n})_{n\in \mathbb{N}}$ as in the previous example, hence Assumption (A1) holds. Rellich’s theorem provides the complete continuity of the embedding

$$ \bigl( W^{1,p}(\Omega ), \lVert \cdot \rVert _{W^{1,p}( \Omega )} \bigr) \to \bigl(W^{1,p}(\Omega ), \lVert \cdot \rVert _{L^{p}(\Omega )} \bigr) $$

which shows Assumption (A4). As for Assumption (A3), Friedrich’s inequality provides the assumptions of Lemma 8. Furthermore, *E* is continuous with respect to $\lVert \cdot \rVert _{W^{1,p}(\Omega )}$ and convex, hence also weakly lower semicontinuous. By Poincaré’s and Young’s inequalities, we find for all $u\in W_{0}^{1,p}(\Omega )$ that

$$\begin{aligned} F^{f}(u) &= \frac{1}{p} \int _{\Omega }\lvert \nabla u\rvert ^{p} \,\mathrm{d}x - f(u) \\ &\geq C \lVert u \rVert ^{p}_{W^{1,p}(\Omega )} - \lVert f \rVert _{W^{1,p}(\Omega )'} \lVert u \rVert _{W^{1,p}( \Omega )} \\ &\geq C \lVert u \rVert ^{p}_{W^{1,p}(\Omega )} - \tilde{C}. \end{aligned}$$

Hence, a minimizing sequence in $W^{1,p}_{0}(\Omega )$ for $F^{f}$ is bounded and as $F^{f}$ is strictly convex on $W^{1,p}_{0}(\Omega )$ it possesses a unique minimizer. Finally, to provide the demicontinuity, we must consider the operator $S\colon W_{0}^{1,p}(\Omega )^{*}\to W_{0}^{1,p}(\Omega )$ mapping *f* to the unique minimizer $u_{f}$ of $E - f$ on $W^{1,p}_{0}(\Omega )$. By the Euler–Lagrange formalism, *u* minimizes $F^{f}$ if and only if

$$ \int _{\Omega }\lvert \nabla u\rvert ^{p-2}\nabla u \cdot \nabla v \,\mathrm{d}x = f(v) \quad \text{for all } v \in W_{0}^{1,p}( \Omega ).$$

Hence, the solution map *S* is precisely the inverse of the mapping

$$ W_{0}^{1,p}(\Omega )\to W^{1,p}_{0}(\Omega )^{*}, \qquad u\mapsto \biggl( v\mapsto \int _{\Omega }\lvert \nabla u\rvert ^{p-2}\nabla u \cdot \nabla v \,\mathrm{d}x \biggr) $$

and this map is demicontinuous, see, for example, Růžička [29].

#### Description of the experiment

**Figure 2:**
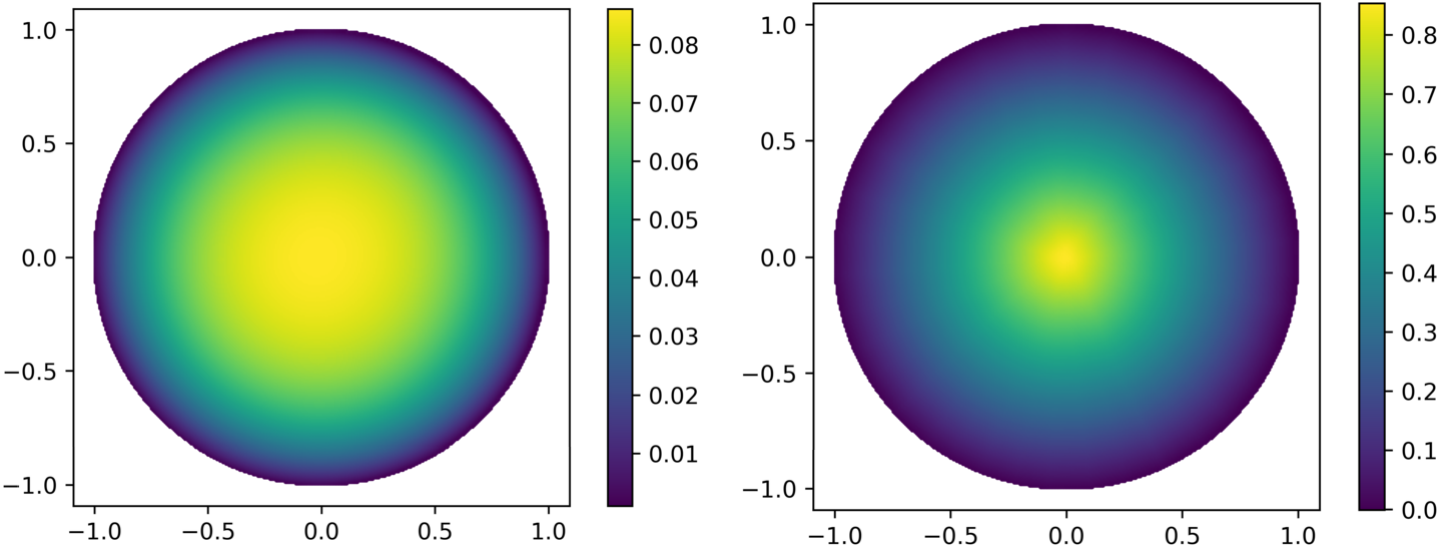
Exemplary numerical realization of the Deep Ritz Method for the *p*-Laplacian with right-hand $f=1$ and $p=1.5$ in the left plot and $p=10$ in the right plot. Zero Dirichlet boundary conditions are enforced through a penalty parameter $\lambda =250$. We used fully connected feed-forward networks with three hidden layers of width 16 and GELU activation for the left plot and ReLU activation for the right plot. The number of trainable parameters is 609. Note the difference in the scaling of the axis in the two plots

Figure 2 shows two numerical realizations of the Deep Ritz Method for the *p*-Laplacian with right-hand side $f\equiv 1$ and $p_{1}=3/2$ in the left picture and $p_{2}=10$ in the right picture. The penalization value is set to $\lambda = 250$ in both simulations to approximately enforce zero boundary values. We used fully connected feed-forward networks with three hidden layers of width 16 and GELU activation (Hendrycks and Gimpel [16]) for the left plot and ReLU activation for the right plot. The quadrature follows the same strategy as in the previous example. Note that the exact solution to the homogeneous *p*-Laplace problem on the disk with $f\equiv 1$ is given by

$$ u_{p}(x) = C\cdot \bigl( 1 - \vert x \vert ^{\frac{p}{p-1}} \bigr) $$

for a suitable constant *C* that depends on the spatial dimension and the value of *p*. We see that the solution $u_{p}$ converges pointwise to zero for $p\searrow 0$ and for $p\nearrow \infty $ the function $u_{p}$ tends to $x\mapsto C(1-|x|)$. This asymptotic behavior is clearly visible in our simulations.

In case of the ReLU ansatz function, the architecture considered agrees with the analysis presented in the previous paragraph. For $p_{1}=3/2$, we found the GELU activation function to provide good performance. However, for the GELU activation function, establishing condition (A1) is not entirely obvious. The GELU activation is defined as $\operatorname{GELU}(x):= x \Phi (x)$, where Φ is the cumulative distribution function of the Gaussian normal. It is often interpreted as a smoothed version of the ReLU since $t^{-1}\operatorname{GELU}(tx) \to \operatorname{ReLU}(x)$ and $\partial _{x} (t^{-1}\operatorname{GELU}(tx)) \to \partial _{x} \operatorname{ReLU}(x)$ pointwise for $t\to \infty $. This gives some intuition why the GELU activation function could admit a universal approximation result with almost zero boundary values and hence satisfy (A1), however, we leave a rigorous statement for future research. This aligns very well with our numerical experiments, which do not indicate problems in resolving the zero boundary values in this practical example.

## Data Availability

The manuscript does not make use of data. Numerical code is not made available.

## Appendix: Universal approximation with zero boundary values

Here we prove the universal approximation result which we stated as Theorem 2 in the main text. Our proof uses that every continuous, piecewise-linear function can be represented by a neural network with ReLU activation function and then shows how to approximate Sobolev functions with zero boundary conditions by such functions. The precise definition of a piecewise linear function is the following.

### Definition 15

(Continuous piecewise-linear function)

We say that a function $f \colon \mathbb{R}^{d} \to \mathbb{R}$ is *continuous piecewise linear,* or in short *piecewise linear,* if there exists a finite set of closed polyhedra whose union is $\mathbb{R}^{d}$, and *f* is affine linear over each polyhedron. Note every piecewise linear functions is continuous by definition since the polyhedra are closed and cover the whole space $\mathbb{R}^{d}$, and affine functions are continuous.

### Theorem 16

(Universal expression)

*Every ReLU neural network function* $u_{\theta}\colon \mathbb{R}^{d}\to \mathbb{R}$ *is a piecewise*-*linear function*. *Conversely*, *every piecewise*-*linear function* $f\colon \mathbb{R}^{d}\to \mathbb{R}$ *can be expressed by an ReLU network of depth at most* $\lceil \log _{2}(d+1)\rceil +1$.

For the proof of this statement, we refer to Arora et al. [2]. We turn now to the approximation capabilities of piecewise linear functions.

### Lemma 17

*Let* $\varphi \in C_{c}^{\infty}(\mathbb{R}^{d})$ *be a smooth function with compact support*. *Then for every* $\varepsilon >0$ *there is a piecewise*-*linear function* $s_{\varepsilon}$ *such that for all* $p\in [1,\infty ]$ *it holds*

$$\begin{aligned} \lVert s_{\varepsilon}-\varphi \rVert _{W^{1,p}(\mathbb{R}^{d})}\le \varepsilon \quad \textit{and}\quad \operatorname{supp}(s_{\varepsilon}) \subseteq \operatorname{supp}(\varphi ) + B_{\varepsilon}(0). \end{aligned}$$

*Here*, *we set* $B_{\varepsilon}(0)$ *to be the* *ε*-*ball around zero*, *i*.*e*., $B_{\varepsilon}(0)=\{ x\in \mathbb{R}\mid \lvert x\rvert < \varepsilon \}$.

### Proof

In the following we will denote by $\lVert \cdot \rVert _{\infty}$ the uniform norm on $\mathbb{R}^{d}$. To show the assertion, choose a triangulation $\mathcal{T}$ of $\mathbb{R}^{d}$ of width $\delta =\delta (\varepsilon ) >0$, consisting of rotations and translations of one nondegenerate simplex *K*. We choose $s_{\varepsilon}$ to agree with *φ* on all vertices of elements in $\mathcal{T}$. Since *φ* is compactly supported, it is uniformly continuous, and hence it is clear that $\lVert \varphi -s_{\varepsilon}\rVert _{\infty }<\varepsilon $ if *δ* is chosen small enough.

To show convergence of the gradients, we show that also $\lVert \nabla \varphi - \nabla s_{\varepsilon}\rVert _{\infty}< \varepsilon $ which will be shown on one element $K\in \mathcal{T}$ and as the estimate is independent of *K* is understood to hold on all of $\mathbb{R}^{d}$. So let $K\in \mathcal{T}$ be given and denote its vertices by $x_{1},\dots ,x_{d+1}$. We set $v_{i} = x_{i+1}-x_{1}$, $i=1,\dots ,d$ to be the vectors spanning *K*. By the one-dimensional mean value theorem, we find $\xi _{i}$ on the line segment joining $x_{1}$ and $x_{i}$ such that

$$ \partial _{v_{i}}s_{\varepsilon}(v_{1}) = \partial _{v_{i}}\varphi ( \xi _{i}). $$

Note that $\partial _{v_{i}}s_{\varepsilon}$ is constant on all of *K* where it is defined. Now for arbitrary $x\in K$, we compute with setting $w=\sum_{i=1}^{d}\alpha _{i}v_{i}$ for $w\in \mathbb{R}^{d}$ with $|w|\leq 1$. Note that the $\alpha _{i}$ are bounded uniformly in *w*, where we use that all elements are the same up to rotations and translations,

$$\begin{aligned} \bigl\lvert \nabla \varphi (x) - \nabla s_{\varepsilon}(x)\bigr\rvert &= \sup _{ \lvert w\rvert \leq 1}\bigl\lvert \nabla \varphi (x)w-\nabla s_{ \varepsilon}(x)w\bigr\rvert \\ &\leq \sup_{\lvert w\rvert \leq 1} \sum_{i=1}^{d} \lvert \alpha _{i} \rvert \cdot \underbrace{ \bigl\lvert \partial _{v_{i}}\varphi (x) - \partial _{v_{i}}s_{\varepsilon}(x) \bigr\rvert }_{=(*)}, \end{aligned}$$

where again $(*)$ is uniformly small due to the uniform continuity of ∇*φ*. Noting that the $W^{1,\infty}$-case implies the claim for all $p\in [1,\infty )$ finishes the proof. □

We turn to the proof of Theorem 2 which we state again for the convenience of the reader.

### Theorem 18

(Universal approximation with zero boundary values)

*Consider an open set* $\Omega \subseteq \mathbb{R}^{d}$ *and let* $u\in W^{1,p}_{0}(\Omega )$ *with* $p\in [1,\infty )$. *Then for all* $\varepsilon >0$ *there exists a function* $u_{\varepsilon}\in W^{1,p}_{0}(\Omega )$ *that can be expressed by an ReLU network of depth* $\lceil \log _{2}(d+1)\rceil +1$ *such that*

$$ \lVert u - u_{\varepsilon } \rVert _{W^{1,p}(\Omega )} \le \varepsilon . $$

### Proof

Let $u\in W^{1,p}_{0}(\Omega )$ and $\varepsilon >0$. By the density of $C_{c}^{\infty}(\Omega )$ in $W^{1,p}_{0}(\Omega )$, see, for instance, Brezis [5], we choose a smooth function $\varphi _{\varepsilon}\in C^{\infty}_{c}(\Omega )$ such that $\lVert u-\varphi _{\varepsilon}\rVert _{W^{1,p}(\Omega )}\leq \varepsilon /2$. Furthermore, we use Lemma 17 and choose a piecewise-linear function $u_{\varepsilon}$ such that $\lVert \varphi _{\varepsilon}-u_{\varepsilon}\rVert _{W^{1,p}( \Omega )}\leq \varepsilon /2$ and such that $u_{\varepsilon}$ has compact support in Ω. This yields

$$ \lVert u-u_{\varepsilon}\rVert _{W^{1,p}(\Omega )}\leq \lVert u- \varphi _{\varepsilon}\rVert _{W^{1,p}(\Omega )}+\lVert \varphi _{ \varepsilon}-u_{\varepsilon} \rVert _{W^{1,p}(\Omega )}\leq \varepsilon $$

and, by Theorem 16, we know that $u_{\varepsilon}$ is in fact a realization of a neural network with depth at most $\lceil \log _{2}(d+1)\rceil +1$. □

## Footnotes

1 and hence also for deep as can be seen by composing with almost identity layers
